# Telomere mean length in patients with diabetic retinopathy

**DOI:** 10.1038/srep18368

**Published:** 2015-12-16

**Authors:** Rupali Sharma, Amod Gupta, M. Thungapathra, Reema Bansal

**Affiliations:** 1 Advanced Eye centre, PGIMER Chandigarh, India.; 2 Department of Biochemistry, PGIMER Chandigarh, India.

## Abstract

Telomere regression has been shown to be associated with several complex disorders like diabetes mellitus, cancer, cataract etc. Diabetic retinopathy develops as a complication of chronic hyperglycemia leading to increased oxidative stress that may potentially lead to shortening of telomeres. We sought to determine whether there is any association between telomere mean length (TML) of peripheral blood monocytes with the presence and severity of diabetic retinopathy. The study involved 120 subjects, comprising 27 non-insulin dependent diabetes mellitus (NIDDM) without any diabetic retinopathy (NDR), 45 NIDDM subjects with non-proliferative diabetic retinopathy (NPDR), 12 NIDDM subjects with proliferative diabetic retinopathy (PDR) and 36 healthy controls. Determination of TML of the study subjects was performed by Southern hybridization using telomere probe. Among the biochemical parameters, HBA1c showed a negative correlation with shortened telomeres in the PDR subjects. However, telomere length was positively correlated with high density lipo protein (HDL) in the control subjects. The control group had significantly greater TML as compared to the rest of the groups and the NDR subjects with NPDR and PDR had substantially decreased TML than the NIDDM subjects without retinopathy.

Telomeres are tandem repeats (TTAGGG)_n_ of the DNA sequences that form a cap at the ends of eukaryotic chromosomes^1^. During somatic cell division telomeres undergo attrition due to the inability of DNA polymerases to fully repair the replication of the 3′-end of a linear DNA molecule^2^. The Nobel Prize winners in Physiology or Medicine in 2009 highlighted how chromosomes are protected by telomeres and the enzyme telomerase, their role in genome stability, immortality, and aging. One way to counteract telomeric attrition is through the activation of the enzyme telomerase, a ribonucleo protein complex that uses its RNA component as a template to add TTAGGG repeats onto the ends of chromosomes^3^,^4^,^5^.

Diabetes mellitus involves microvascular (diabetic nephropathy, neuropathy, and retinopathy) and macrovascular (coronary artery disease, peripheral arterial disease, and stroke) complications. Patients with diabetes mellitus are at a higher risk for micro and macrovascular diseases^6^, one of the most devastating being diabetic retinopathy (DR). Diabetic retinopathy develops as a complication of chronic hyperglycemia and is the main cause of vision impairment in these patients.

Diabetic patients having shorter telomeres^7^,^8^,^9^,^10^ but good glycemic control has been associated with favorable telomere dynamics^11^,^8^. Telomere attrition has also been shown to be associated with cardio vascular disease and peripheral vascular disease^12^,^13^. With this background, we proposed to study the telomere length in diabetic subjects in various stages of retinopathy.

## Results

**Figure 1:**
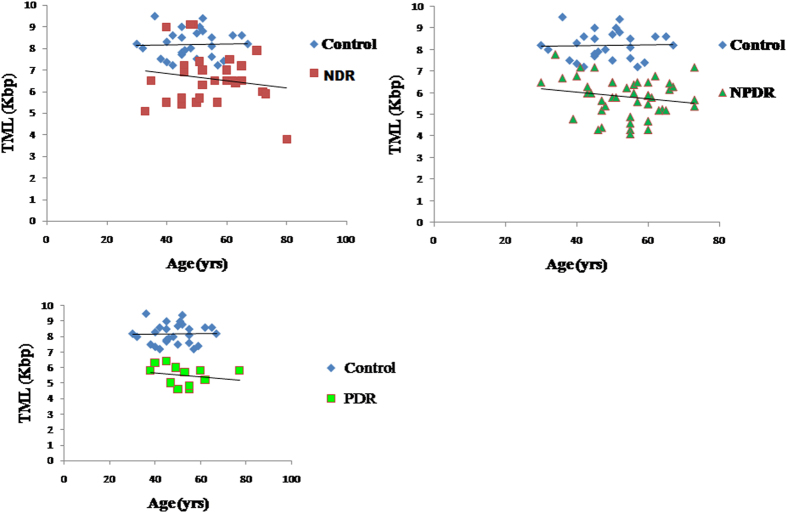
Regression plots showing TML of various groups 1) Control and NDR subjects 2) Control and NPDR subjects 3) Control and PDR subjects.

**Figure 2:**
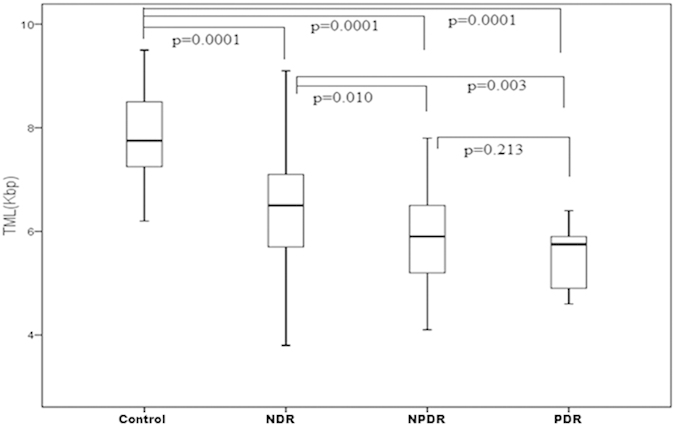
Box plot showing telomere mean length (TML) among various groups. (Control group had significantly higher TML as compared to the rest of the groups (p < 0.05) and that of NDR was significantly higher as compared to NPDR (p = 0.010) and PDR (p = 0.003).

**Figure 3:**
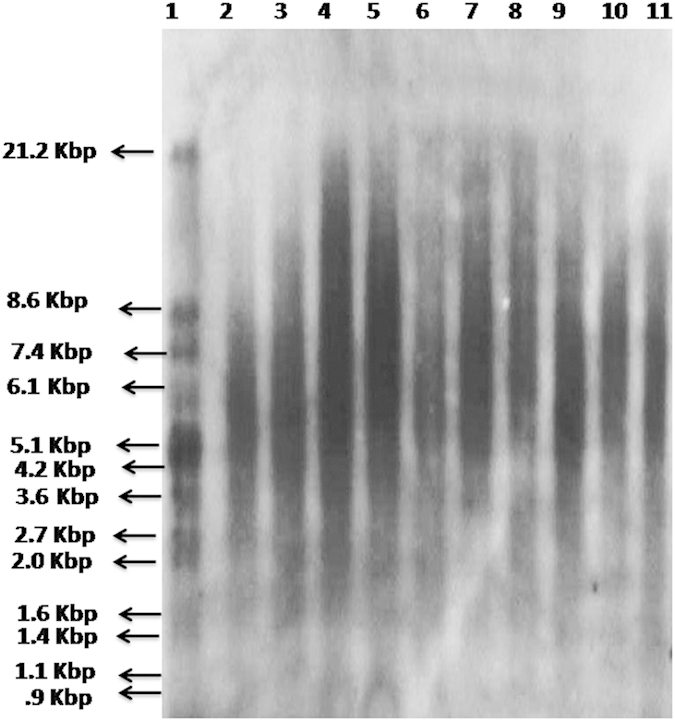
Distribution of TRFL’s in PBMC’s. Lane 1: DIG Molecular weight marker. Lane 2–11: Chemiluminescence of the digested DNA samples.

Telomere length has been correlated with biological aging and its attrition has been associated with number of diseases like diabetes and hypertension. The logistics of doing this study is very clear as hyperglycemia leads to increased oxidative stress, thus resulting in the accelerated aging of somatic cells. Increased oxidative stress and insulin resistance may also be involved in the pathogenesis of the most devastating complication of diabetes which is DR. Thus we sought to analyze the telomere length which is affected by oxidative stress in a number of disorders and if it can be used as a prognostic marker for DR. The telomere mean length was determined in 120 subjects. Subjects enrolled were free from any other disorder such as obesity and cardiovascular disorder. There were 36 healthy controls (age range: 30 to 67 years), 27 NDR patients (33 to 80 years), 45 Mild NPDR patients (30 to 73 years) and 12 PDR patients (38 to 77 years). Differences in age at blood collection reflect the age-based definitions of NDR, NPDR and PDR and the recruitment of adult control subjects of all ages. Besides age the other biochemical parameters included Hb, HbA1c, FBS, TC, TG, HDL,VLDL, BUN, creatinine, TP, FI, IR, systolic and diastolic blood pressure measurements (Table 1). Among the biochemical parameters, HbA1c levels showed a negative correlation with shortened telomere length in the PDR subjects (r = −0.695, p = 0.012) clearly indicating that hyperglycemic state is associated more closely with the telomeric shortening. However, telomere length was positively correlated with HDL (r = 0.353, p = 0.035) in the control subjects indicating that HDL exerts anti-oxidant and anti-inflammatory effects in the control group. Figure 1 depicts the relationship between TRF length and age at the time of blood donation. We saw a clear regression in the telomere length with age in the NDR, NPDR and PDR groups in comparison to the control group. A slight decreasing trend was also observed in control group though not to an extent the way we observed in the NDR, NPDR and PDR groups because all these groups are diabetic. Another important thing should be kept in mind that due to diabetes many metabolic changes takes place in the cell leading to more oxidative stress which may be the cause of more regression of telomere length in the diseased groups in comparison to the control when age matched. Figure 2 depicts the box plots showing telomere length in various groups. Control group had significantly higher TML in comparison to the NDR, NPDR and PDR group (p = 0.0001), NDR group had significantly higher TML in comparison to the NPDR and PDR group (p = 0.010 and p = 0.003 respectively). We could not see significant changes in the TML length in the PDR group in comparison to the NPDR group because of the less number of subjects in the PDR group. The box plot in Fig. 2 depicts the difference in the TML between normal and diabetic subjects with and without retinopathy individuals. Figure 3 shows the distribution of TRFLs in peripheral blood monocytes (PBMCs) after chemiluminescence. Table 2 shows that the control group had significantly higher TML as compared to the rest of the groups (p < 0.05) and that of NDR was significantly higher as compared to NPDR (p = 0.010) and PDR (p = 0.003). The inter quartile range (IQR) of the TML in the control group is itself high in comparison to all diseased groups.

## Discussion

This is the first study reporting an association between DR and telomere length in human subjects. Telomere length is a novel biomarker of physiologic aging and its biology has been the focus of numerous investigations. Many of these have attempted to understand the role of telomeres in human cancer^14^,^15^, hypertension and atherosclerosis^16^. Recently, it was shown that patients with diabetes had more dramatic vascular changes in comparison to the control group resulting in shorter telomeres^17^. Keeping all this in mind we hypothesized that there may be a connection between telomere length and diabetic retinopathy.

Longer duration and poor control of diabetes mellitus is associated with advancing stages of DR. Telomere length reflects cellular turnover and exposure to oxidative and inflammatory damage^18^. In our study, telomere length decreased with age in both the patients and healthy controls but remained shorter in diabetic subjects as compared to healthy controls (even after adjustment for age). The difference between patients and controls also persisted after stratification into five age groups, indicating that diabetes and DR influenced telomere length at all ages. Our results are in accordance with the results of others who found that the mean length of telomeres (expressed as the TRF length) of somatic cells is progressively shortened as a function of the donor’s age^19^,^20^,^21^,^22^. In this study we also found significant difference between the mean TRF of PBMCs from diabetic individuals (with and without retinopathy) and control subjects, and this difference was greater for the group with retinopathy as compared to the diabetic group without any retinopathy. The results of our study suggest that TRF in patients with DR is related to severity of the disease. Oxidative stress in various disorders like diabetes has become an increasingly prominent marker of aging^18^ leading to shorter telomere length. While in NPDR and PDR there is increased inflammation which may also be one of the cause of shortening of TRF length. Association with telomere length have been reported in essential hypertension^23^,^1^, diabetes^9^, insulin resistance^18^, obesity^23^, atherosclerosis^24^,^25^,^26^, vascular dementia^27^, and mortality due to heart disease^27^. Till now significant differences have been found between the mean telomere length from PBMCs of diabetic individuals and normal control subjects.

The main strength of the current study is that it is the first to compare the telomere length in various stages of DR. We found that the association between decreased TML and increasing age was stronger in subjects with DR indicating that subjects presenting with the coexistence of DR and increasing age were more likely to have increased risk of shortened TML in comparison to age matched NDR. It has been reported that 80 percent of all patients who had diabetes for 10 years or more were more prone to DR. If TML is apparently shorter in patients with diabetes when age matched with other diabetics then in this case TML can be used as one of the biomarker or risk factor for DR. In this way new cases of DR can be reduced with proper prognosis and vigilant treatment and monitoring of the eyes. A clear regression in the telomere length with age in the NDR, NPDR and PDR groups in comparison to the control group was observed. Another important thing should be kept in mind is that a slight decreasing trend was observed in the control group though not to an extent the way we observed in the NDR, NPDR and PDR groups because all these groups are diabetic and due to diabetes many metabolic changes takes place in the cell leading to more oxidative stress which may be the cause of more regression of telomere length in the diseased groups in comparison to the control when age matched. So we can say that the affect what we see in TML is due to DR in accordance to age. It is already known that patients with type 2 diabetes have shorter telomeres than the non-diabetics^10^,^28^ but this is for the first time that we found significantly shorter mean TRF length among diabetic (NIDDM) subjects having no DR (NDR) and subjects having DR in different stages i.e NPDR and PDR. Among the biochemical parameters HbA1c showed a negative correlation with shortened telomeres in the PDR subjects. In a study by Tamura *et al.* they found an inverse correlation between HbA1c and TML in the diabetic subjects^29^. Significant positive correlation with HDL in the control subjects is noteworthy. However, several limitations should be considered in the interpretation of our results. First, the association reported in this study is in a very small number of subjects so larger study samples are required to throw more insight into the association of telomere length with DR and establish the fact of using TML as a prognostic marker for DR. Thus, the results should be regarded as preliminary and further studies are needed to establish this fact considering large number of samples in each group.

## Material and Methods

### Study subjects

This study included 120 subjects visiting the Department of Ophthalmology, Advanced Eye Centre, Post Graduate Institute of Medical Education and Research, Chandigarh, India, from August 2008 to July 2011. The study was approved by the Institute Ethics Committee and informed consent was obtained from patients. All methods were carried out in accordance with the approved guidelines. Selection and enrollment of patients was based on detailed ocular examination including estimation of best corrected visual acuity (BCVA), intraocular pressure and dilated fundus examination. Patients were categorized into No retinopathy (NDR), Non Proliferative diabetic retinopathy (NPDR) and proliferative diabetic retinopathy (PDR) based on Early Treatment Diabetic Retinopathy Study (ETDRS) classification.

### Selection and enrollment of patients

Patients were questioned about previous and current diseases, use of medications and their smoking habits. Ex- smokers who had given up smoking for a period of at least 3 years were considered. Patients diagnosed as having Non-Insulin Dependent Diabetes Mellitus (NIDDM) (any duration) were enrolled in the study. NIDDM was diagnosed if diabetes was diagnosed at age 26 years or older and the patient was not treated with insulin until at least 1 year after diagnosis. The inclusion criteria of patients assigned to various groups were following:

**Group A (Healthy controls n = 36).** This group included subjects who visited the hospital for routine eye evaluation. These subjects had no history of any systemic disease such as diabetes, hypertension, etc. The ophthalmic evaluation in them was unremarkable and the BCVA was 6/6 on Snellen’s visual acuity chart. These subjects had normal levels of fasting blood sugar, haemoglobin (Hb), HbA1c and serum lipid profile. **Group B (NDR n = 27).** This included patients with NIDDM. They had fasting blood sugar above the normal range but had no features of DR on detailed fundus examination. **Group C (NPDR n = 45).** Patients with NIDDM (fasting blood sugar above the normal range) who had various features of Non Proliferative diabetic retinopathy (NPDR) on dilated fundus examination. **Group D (PDR n = 12)**. This group had NIDDM patients who had evidence of proliferative diabetic retinopathy (PDR) in one or both eyes. In cases of asymmetrical DR between the two eyes of a patient, presence of the more advanced form of DR determined the group of that patient. Patients with any coexisting ocular disease such as glaucoma, media opacities, retinal vascular occlusions, ocular trauma or intraocular surgery were excluded from the study.

### Collection of blood samples

Blood was collected after an overnight fast of at least 12 hours. About 10 mL of whole blood was collected from each patient and split into heparinized (about 9 mL) and unheparinized (1 mL) vials. Unheparinized blood was used to carry out Hb and HbA1C tests. From the rest, serum was separated for biochemical analysis.

### Biochemical analysis

Serum glucose, lipids (total cholesterol, HDL cholesterol, and triglycerides) and creatinine were measured enzymatically on an auto analyzer (Erba Chem7). LDL cholesterol was calculated using the formula of Fried Wald *et al.*^30^ HbA1C was measured by using resin exchange column technique. Plasma insulin concentrations were measured by homeostasis model assessment and were used to calculate insulin resistance (HOMA-IR) so as to choose proper control subjects.

### Isolation of genomic DNA from peripheral blood monocytes (PBMCs)

Briefly, heparinized blood was processed within 2 hours of collection. PBMCs were separated from whole blood diluted with 1X PBS (1:1) by centrifugation (@1,500 rpm for 30 min at room temperature) on a ficoll gradient (1.077 g/ml, HIMEDIA, India). The PBMCs present in the interphase as a white ring between plasma phase and the ficoll, were carefully removed. PBMCs were washed in phosphate-buffered saline (1XPBS), centrifuged (3,000 rpm, 5 min) and the cell pellet was further used for DNA isolation^31^.

### TRF length measurement

Telomere length analysis was performed by the TeloTAGGG telomere length assay kit (Roche, Germany) as described by the manufacturer. Briefly, ten DNA samples (~1.5 μg each) and two DNA ladders (DIG- Molecular weight marker, Roche, Germany) and λ DNA fragments digested with HindIII (0.3 μg/μL,Fermentas Life Science, USA) were digested overnight with restriction enzymes HinfI and RsaI (40 U/μL Roche,Germany) and resolved on a 0.8% agarose gel (7 cm  × 10 cm) at 40 V (PowerPac Basic, Bio-Rad). Southern transfer of the separated DNA was carried out essentially as described^32^. The blot was first prehybridized before hybridization with the telo probe (digoxigenin 3′-end labeled 5′-(CCCTAA)_3_) as recommended by the manufacturer. After washing the unbound probe and blocking the membrane, the blot was treated with anti DIG antibody and developed with the substrate. The digoxigenin labeled probe bound to the membrane was detected by the digoxigenin luminescent detection procedure (Roche) and exposed on X-ray film. The mean TRF of every sample was determined from the autoradiogram by comparison with the molecular size markers. All lanes were subdivided into intervals of ~1 ± 2 mm. The mean size of the TRF was estimated using the formula €(ODi ± background)/€(ODi ± background/Li)^33^ where ODi is the chemiluminescent signal and Li is the length of the TRF fragment at position i. The TRF length measurements was completely blinded to all characteristics of participants.

### Statistical analysis

In order to see whether the data was normally distributed, normal-quartile (Q-Q) plots were constructed. As our data was skewed we calculated median and inter quartile range. To compare the groups, non parameteric test Kruskal Wallis H was applied followed by Mann Whitney U statistics^34^. All statistical values were two sided and p ≤ 0.05 was considered to be significant. Statistical analysis was performed with the help of SPSS 20 software.

## Additional Information

**How to cite this article**: Sharma, R. *et al.* Telomere mean length in patients with diabetic retinopathy. *Sci. Rep.* **5**, 18368; doi: 10.1038/srep18368 (2015).

## Figures and Tables

**Table 1:** Median (IQR) of various parameters for control and NIDDM subjects with and without retinopathy.

Parametrs	Control (n = 36)	NDR (n = 27)	NPDR (n = 45)	PDR (n = 13)
Age (yrs)	45 (38.5–51.75)	52 (46–63)	55 (46.5–60.5)	51.50 (45.50–58.75)
Hb	12.48 (11.40 13.73)	12.7(11.2–14.4)	13.2 (11.45–14.30)	12.05 (11.13–13.75)0.712*, 0.009**
HbA1c	4.80 (4.3–5.2)	6.72 (6.29 8.15)	6.90 (6.38–7.55)	6.95 (6.38–7.58) −695*, 0.012**
FBS	92.25 (83.52–97.9)	148.2 (129–189)	149.8 (110.75–184.75)	137.05 (95.64–177.22)
TC	165.5 (151–187.5)	175 (129.4–205)	174.6 (143.4–192.22)	166.55 (128.8–247.72)
TG	139.5(100–155.5)	112(80.2–138.2)	101.6 (80.29–152.6)	121.05 (85.52–195.25)
HDL	42.15 (38.35–46) 0.353*, 0.035**	45 (35.6–52)	45.3 (39.35–55.38)	40.62 (38.16–48.71)
LDL	91.8 (86.3–109.5)	108 (67.84–132.6)	95.9 (74.16–120.56)	94.69 (69.81–157.86)
VLDL	27.9 (20–31.1)	22.40 (16.04–27.64)	20.32 (16.06–30.52)	24.21 (17.10–39.05)
BUN	10 (9.65–12)	17 (13–20)	18 (14–24.5)	22.50 (18–42.50)
Creatinine	0.96 (0.89–1.07)	0.94 (0.80–1.11)	1.17 (94–1.32)	1.04 (0.95–1.55)
TP	7.15 (6.65–7.3)	7.3 (6.68–7.58)	7.3 (6.96–7.70)	7.16 (6.77–7.63)
FI	10.12 (9.17–12.93)	9.87 (7.49–12.49) 0.423*, 0.028**	12.62 (9.17–19.24)	12.82(9.73–16.19)
IR	2.47 (2.01–3.41)	3.55 (2.71–6.53)	4.18 (2.74–7.7)	4.77 (2.29–6.55)
SBP	120 (116–130)	130 (120–150)	140 (120–155)	135 (110–143.75)
DBP	80 (75–90)	90 (80–90)	90 (80–90)	82.5 (75–90)

Data are median (interquartile range), Correlation coefficients (*) and p value (**) of telomere length with classical risk factors in control subjects and NIDDM subjects with and without retinopathy.

TML in relation to Hb (r = 0.712, p = 0.009) and HbA1c (r = −0.695, p = 0.012) in the PDR subjects, HDL (r = 0.353, p = 0.035) in control subjects, FI (r = 0.423, p = 0.028) in NDR subjects.

Haemoglobin (Hb), Glycosylated Hemoglobin type A1C (HbA1c), Fasting blood sugar (FBS), Total cholestrol (TC), Triglyceride (TG), High density lipoprotein (HDL), Low density lipoprotein (LDL),Very low density lipo protein (VLDL), Blood urea Nitrogen (BUN), Total Protein (TP), Fasting Insulin (FI), Insulin Resistance (IR), systolic blood pressure (SBP), Diastolic blood pressure (DBP).

**Table 2:** Median (IQR) of telomere mean length for various groups.

Groups Total (n = 120)	Median (IQR)
Control (n = 36)	7.75 (7.22–8.50)
NDR (n = 27)	6.50 (5.70–7.20)
NPDR (n = 45)	5.90 (5.20–6.50)
PDR (n = 12)	5.75 (4.85–5.95)

(IQR = Interquartile range, NDR = Diabetic with no Diabetic retinopathy, NPDR = Diabetic with non-proliferative diabetic retinopathy, PDR = diabetic with proliferative diabetic retinopathy).
